# Cognitive Behavioral Therapy for Insomnia in Breast Cancer Survivors: A Review of the Literature

**DOI:** 10.3389/fpsyg.2016.01162

**Published:** 2016-08-03

**Authors:** Debora Aricò, Alberto Raggi, Raffaele Ferri

**Affiliations:** ^1^Department of Neurology, Oasi Institute for Research on Mental Retardation and Brain AgingTroina, Italy; ^2^Unit of Neurology, G. B. Morgagni- L. Pierantoni HospitalForlì, Italy

**Keywords:** breast cancer survivors, cognitive behavioral therapy for insomnia, health-related quality of life, insomnia, mood, oncology, review

## Abstract

**Background:** Insomnia is a common sleep disorder in patients with breast cancer and studies show a higher frequency than in the general population but it appears to be understudied and the treatment seems to be a neglected problem. There is a growing body of evidence about the efficacy of cognitive behavioral therapy for insomnia (CBT-I) in breast cancer survivors (BCS). The aim of this review is to examine the best available scientific evidence related to CBT-I and insomnia in patients with breast cancer and to assess the effect of CBT-I on their psychosocial functioning, sleep, quality of life, and mood.

**Methods:** Empirical articles published in peer-reviewed journals from the earliest reports available until August 2015 were considered. The research on PubMed generated 18 papers, three of which did not meet the inclusion criteria. Another paper was retrieved by screening the reference list of the previously selected papers.

**Results:** A total of 16 studies were found that evaluated the effects of CBT-I in breast cancer patients. CBT-I appears to be an effective therapy for insomnia in BCS, improving mood, general and physical fatigue, and global and cognitive dimensions of quality of life. CBT-I may also reduce menopausal symptoms, such as hot flushes and night sweat problems, frequency of medicated nights, level of depression, and anxiety.

**Conclusions:** CBT-I seems to be an eligible intervention for improving sleep in BCS. Improvements concerning insomnia and sleep quality are durable (usually up to 12 months) and statistically significant.

## Introduction

Insomnia is defined by the International Classification of Sleep Disorders, third edition (American Academy of Sleep Medicine, 2014) as “a persistent difficulty with sleep initiation, duration, consolidation, or quality that occurs despite adequate opportunity and circumstances for sleep, and results in some form of daytime impairment.”

Insomnia is characterized by subjective complaints about dissatisfaction with sleep quality or duration, difficulty falling asleep at bedtime, waking up too early in the morning or in the middle of the night, or non-restorative, or poor quality sleep. Insomnia also involves subjective reports of daytime symptoms such as fatigue or low energy, difficulties with cognitive functions, for instance attention, concentration, and memory, and mood disturbances including irritability and dysphoria, all of which can produce functional impairment and are often the primary concerns pushing patients to seek treatment.

Patients with the diagnosis of breast cancer report very often insomnia (Ohayon, 2002). It has been estimated that ~30–60% of breast cancer women experience insomnia and the prevalence is higher than in non-cancer patients (Savard et al., 2009; Palesh et al., 2010). Notwithstanding the wide spread of the phenomenon in this population, insomnia is largely understudied. It has a high prevalence in women with breast cancer for several reasons, such as a general increase in psychological distress after the cancer diagnosis and disruption of sleep due to increased frequency and severity of hot flushes caused by menopause, often induced by the chemotherapy (Fiorentino et al., 2010). Insomnia seems to be determined by predisposing, precipitating, and perpetuating factors (Spielman et al., 1987; American Psychiatric Association, 1994; Bastien et al., 2004). Predisposing factors can be several such as gender, older age, hyperarousability as a trait, personal or family history, mood or trait anxiety, predisposition to rumination; precipitating factors consist of diagnosis of cancer, severity of disease, cancer treatment that alter the levels of inflammatory cytokines or disrupt circadian rhythms or sleep-wake-cycles, side effects of cancer treatment, menopausal symptoms including pain or fatigue, and medications used to treat side effects such as corticosteroids. Moreover, perpetuating behavioral factors such as long term use of medications or use of inappropriate medications, and maladaptive coping, i.e., inaccurate appraisal of sleep difficulties and quality (American Psychiatric Association, 1994; Bastien et al., 2004).

Cognitive behavioral therapy for insomnia (CBT-I) is a brief, sleep-focused, multimodal intervention (Morin and Espie, 2003; Edinger and Carney, 2008). CBT-I is considered to be the gold standard treatment for primary and comorbid insomnia in young and older adults (Morin and Benca, 2012). The most common approach includes a behavioral component (stimulus control, sleep restriction, relaxation) combined with a cognitive and an educational component (sleep hygiene). Each of these therapies can be used alone or in combination; however, the combined approach is preferred because several dimensions of insomnia can be addressed at the same time.

An increasing number of studies have supported the efficacy of CBT-I in patients with cancer (Garland et al., 2014). Overall, results have been quite consistent in showing that CBT-I is associated not only with improved sleep but also with a reduction of psychological distress and improved quality of life (QoL). This paper reports a review of the literature on CBT-I in breast cancer survivors (BCS) with the aim to assess the effects of CBT-I on psychosocial, sleep, health-related QoL, and mood.

## Materials and methods

Data for this review consisted of empirical articles published in peer-reviewed journals from the earliest data available until August 2015. An extensive computer-assisted literature search was conducted using the PubMed database. Search terms included *Breast Cancer, Sleep* and Sleep Psychotherapy terms such as *cognitive behavior therapy, cognitive behavioral therapy, cognitive behavioral therapy*. Searches were conducted using all possible combinations of sleep and sleep psychotherapy terms and breast cancer. Excluded from consideration were non-English articles, book chapters, monographs, commentaries, review articles, case studies, dissertations, abstracts, letters to the editor, and any non data-analytic or non peer-reviewed reports. However, in the case of review articles, their reference list was used to find additional eligible articles.

The research on PubMed “breast cancer” AND (“cognitive behavior therapy” OR “cognitive behavioral therapy” OR “cognitive behavioral therapy”) AND “sleep” generated 18 papers. They were filtered and three of them were excluded: a review (Mustafa et al., 2013); a study (Mustian, 2013) where CBT-I was not the first line treatment (yoga vs. unspecified standard care treatment), and a paper about male patients with prostate cancer (Yousaf et al., 2012).

The reference list of the retrieved articles was reviewed and articles meeting the following criteria were included: (a) the target population were women diagnosed with breast cancer and insomnia, and (b) cognitive behavioral therapy or behavior therapy tailored for insomnia was used as the primary psychotherapy treatment. This screening produced one additional article (Roscoe et al., 2015) that was added to the final list which included 16 papers in total.

## Results

The studies investigating CBT-I in patients with breast cancer identified for inclusion are listed in Table 1. All of these studies were carried-out in non-metastatic cases (stages 0-III) who had completed treatment for cancer at least 1 month before. The sample size in the studies ranged from 10 to 242 and the majority (75%) used a randomized controlled design. All of them specified treatment status (post radiation, chemotherapy, surgery, life-time, or current hormone therapy), with the exception of two reports: one described generic “post primary treatment” (Dirksen and Epstein, 2008) and the other did not specify it at all (Hunter et al., 2004). Nine studies did not include objective measures because the authors used sleep diaries, questionnaires, self-reported scales, or semi-structured interview to evaluate sleep habits, sleep features, and complaints about sleep. The remaining seven studies used objective measures such as polysomnography (PSG; four reports) or actigraphy (three papers). Individual CBT-I was delivered in only four studies; in the majority of studies (nine) the treatment consisted of group-administered sessions; finally, in three reports this aspect was not specified. Almost in all of the studies, the investigators enrolled patients with chronic insomnia judged to be secondary to the neoplastic condition (insomnia started or worsened significantly at the time of the diagnosis of breast cancer).

**Table 1:** **Summary of the 16 studies investigating CBT-I in patients with breast cancer which were identified for inclusion in the review**.

**Paper**	**Patients**	**Controls**	**Age, years mean ±*SD* (range)**	**Stage**	**Design**	**Treatment**	**Methods**	**Main Findings**
Fiorentino et al., 2010	11	10	61 ± 11.6 (45–85)	Stage I–III	Randomized controlled crossover pilot study	Surgery chemotherapy radiation	Individual CBT-I, delayed treatment control condition	Self-rated insomnia significantly improved. Individual CBT-I beneficial in improving self-rated insomnia, sleep quality, and objective measures of sleep
Roscoe et al., 2015	96	Unspecified	Mean 56	Unspecified (and other types of unspecified cancer)	Randomized placebo-controlled trial	Chemotherapy radiotherapy	CBT-I, CBT-I + armodafinil, placebo, armodafinil	Durable improvements in insomnia and sleep quality with CBT-I. Pharmacological therapy did not increase the efficacy of CBT-I
Dirksen and Epstein, 2008	34	38	57.2 ± 9.9 (29–74)	Stage I–III	Randomized experimental design		CBT-I and component control	CBT-I associated with decrease of fatigue, trait anxiety, depression and improvement in quality of life. Component control group with increase in quality of life (lower depression at post-treatment)
Hunter et al., 2004	113	0	56.3 ± 7.0 (38–65)		Cross-sectional descriptive study		Questionnaires, semi-structured interviews, treatment preferences for menopausal symptoms	Non-medical treatments preferred, particularly vitamins and herbal remedies, and CBT-I. CBT-I reduced menopausal symptoms
Quesnel et al., 2003	10	0	54.3 ± 7.5 (39–67)	Stage I–III	Multiple baseline design	Radiotherapy chemotherapy	Group-administered therapy	Improvement in mood, general and physical fatigue, and global and cognitive dimensions of quality of life.
Epstein and Dirksen, 2007	34	38	57.1 ± 9.8 (29–74)	Stage I–III	Randomized controlled design	Surgery radiotherapy chemotherapy	Multicomponent intervention group and component control group (sleep education, hygiene)	Both groups showed significant changes on sleep-onset latency, wake after sleep onset, total sleep time, time in bed, sleep efficiency, and sleep quality based on sleep daily dairies. When compared to the control group, multicomponent intervention group rated overall sleep as more improved.
Tremblay et al., 2009	27	30	54.05 ± 7.36	Stage I–III	Randomized controlled design	Radiotherapy chemotherapy lumpectomy/mastectomy hormones	CBT-I and waiting-list control condition	Levels of expectancies and low dys-functional beliefs about sleep predicted subjective response to CBT-I, at post-treatment. Low dysfunctional beliefs and avoidance of day napping predicted PSG improvement.
Savard et al., 2011	11	0	Mean 51.5 (37–74)	Stage I–III	Single group design	Radiotherapy	VCBT-I, semi-structured interview, self-report scales, daily sleep diary	High adherence rates and patient satisfaction.
Rumble et al., 2010	41	0	57 ± 8.2	Stage I–III	Within-group longitudinal research design	Surgery chemotherapyradiotherapy	28-day (morning and evening diary)	Relationship between poorer sleep, pain and hot flushes. Dysfunctional sleep related thoughts and sleep inhibitory behaviors during the day and night antecedents of insomnia. Poorer sleep significantly predicting higher levels of pain, fatigue and hot flushes and lower levels of positive mood the next day
Mann et al., 2011	96	0			Randomized controlled design	Surgery radiotherapy chemotherapy	CBT-I/usual care	CBT-I effective to reduce hot flushes and night sweats
Mann et al., 2012	96	0			Randomized controlled design	Chemotherapy radiotherapy surgery	CBT-I/usual care	CBT-I effective for hot flushes, night sweats, mood, sleep, and quality of life
Savard et al., 2005a	27	30	54.1 ± 7.4	Stage I–III	Randomized controlled design	Radiotherapy chemotherapy	CBT-I administered immediately, waiting list control condition	Higher secretion of IFN-γ and lower increase of lymphocytes with CBT-I. Significant changes in WBCS, lymphocytes, and IFN-γ at follow-up compared with post-treatment
Savard et al., 2005b	27	30	54.8 ± 7.0	Stage I–III	Randomized controlled design	Radiotherapy chemotherapy	CBT-I administered immediately, waiting list control condition	CBT-I improved subjective sleep indices (daily sleep diary and insomnia severity index), reduced frequency of medicated nights, levels of depression and anxiety, and improved global quality of life
Matthews et al., 2012	34	24	53.56 ± 7.09 (21–65)	Stage I–III	Randomized controlled design	Chemotherapy radiotherapy lumpectomy/mastectomy, hormones	CBT-I/attention control placebo	CBT-I improved sleep outcomes (nocturnal awakenings). Patient adherence constant throughout CBT-I
Savard et al., 2001	161	81	Mean 54.4 (18–75)	Stage 0–III	Randomized controlled design	Radiotherapy	VCBT-I vs. CBT-I	CBT-I and VCBT-I associated with subjective sleep improvement. CBT-I with greater improvement in insomnia.
Matthews et al., 2014	32	28	(21–65)	Stage I–III	Prospective, longitudinal, randomized, controlled study	Chemotherapy radiotherapy lumpectomy/mastectomy hormones	CBT/behavioral placebo treatment	Nurse-delivered CBT-I improved sleep latency/efficiency, insomnia severity, functioning sleep knowledge and, with sustained benefit over time

CBT-I, cognitive behavioral therapy for insomnia; VCBT-I, video based cognitive behavioral therapy; PCBT-I, professionally administered format cognitive behavioral therapy; IFN-γ, interferon-γ.

### Studies focused on the effects of CBT-I on sleep

Most of the papers focused just on the effects of CBT-I on sleep. In the first study, Quesnel et al. (2003) investigated the efficacy of CBT-I in patients with BCS and chronic insomnia. This study consisted of two phases. In the first phase, patients filled out the sleep diary daily, in the second phase patients underwent to group-administered CBT-I; PSG was recorded before and after the treatment. The study included a follow-up period of 2 weeks after the end of the treatment. In order to evaluate the maintenance of the therapeutic gains, additional evaluations were carried-out 3 and 6 months later. The analysis of sleep diaries showed a progressive reduction in Total Wake Time (TWT) and a progressive increase in Sleep Efficiency (SE), following CBT-I. The daily variation in sleep after treatment was much smaller than at baseline and the therapeutic gains were generally well maintained after treatment. Data from PSG confirmed the results obtained by sleep diaries, highlighting a significant reduction of TWT (after treatment and follow up at 3 and 6 months) and a post-treatment increase in SE.

In order to determine the efficacy of CBT-I in BCS, Epstein et al. (Epstein and Dirksen, 2007) performed a randomized trial with women who completed primary cancer treatment. The study procedure consisted of 2 consecutive weeks of pre-treatment in which all participants completed daily sleep diaries and wore a wrist actigraph each night. The women were randomly assigned to a multicomponent intervention group (stimulus control, sleep restriction therapy, sleep education, and hygiene) or control group (sleep education and hygiene alone). During the 2 weeks of the post-treatment phase, all participants wore the wrist actigraph each night, completed the daily sleep diaries and a questionnaire similar to the pre-treatment instruments. Sleep diary data analysis revealed a significant time effect on sleep-onset latency, Wake After Sleep Onset (WASO), Total Sleep Time (TST), time in bed (TB), sleep efficiency (SE), and sleep quality, indicating that both groups improved after treatment. The sleep diary results showed also that participants of the multicomponent intervention group spent less time in bed than those of the control group after treatment.

Tremblay et al. (2009) investigated the predictors of the effect of CBT-I for chronic insomnia in BCS. The patients were randomly assigned to CBT-I or waiting list. The initial level of expectations and perceived credibility of the treatment were the variables most consistently associated with improvement of subjective sleep variables at post-treatment, whereas improvement of subjective sleep was not associated with PSG-assessed enhancements. At 6-month follow-up, subjectively assessed sleep improvements were best predicted by adherence to the behavioral strategies, but none of the predictors was significantly associated with PSG assessed sleep improvements.

In another study (Fiorentino et al., 2010), the effects of individual CBT-I on sleep were evaluated in a sample of women with a history of breast cancer. The study considered two conditions: treatment condition or delayed treatment control condition. Objective measures of sleep were derived by actigraphy, subjective data were obtained by compiling sleep diaries daily. Self-rated insomnia was significantly improved in the treatment group, when compared to the control group.

In a research on the feasibility of a self-help treatment for insomnia associated with a neoplastic condition, Savard et al. (2011) indicated that self-help CBT-I is a feasible intervention in BCS. Indeed, self-reported adherence levels with specific strategies for insomnia were very high and there was a significant time effect on the Insomnia Severity Index (ISI) total score and on all sleep variables derived from the sleep diaries, QoL, and dysfunctional beliefs and attitudes about sleep scores. Therapeutic effects were maintained at a 3-month follow up.

In a recent study, Roscoe et al. (2015) wondered whether providing breast cancer patients with the wakefulness-promoting agent armodafinil would result in a greater adherence to CBT-I instructions. Patients were randomly assigned to one of four groups: CBT-I, armodafinil, CBT-I plus armodafinil, or no treatment. Both the CBT-I+armodafinil and the CBT-I+placebo groups had significantly greater post-intervention reductions in insomnia severity than the placebo group. There were similar improvements for sleep quality. Therapeutic gains on both measures persisted 3 months later. CBT-I+ armodafinil was not significantly different from CBT-I+placebo.

### Studies focused on the effects of CBT-I on menopausal symptoms

Hunter (Hunter et al., 2004) investigated the experience of menopausal symptoms, the prevalence and treatment preferences in a sample of women who had been prescribed tamoxifen within the past 5 years. The participants completed several questionnaires about types of treatment used to deal with menopausal symptoms and treatment preferences. High rates of symptoms were reported by patients. The majority of women attributed their menopausal symptoms to tamoxifen. Nevertheless, the adherence to treatment with tamoxifen remained generally very high. Levels of depressed mood were reported to be significantly higher than an equivalent non-breast cancer, mid-aged sample. The experience of hot flushes was not associated with depressed mood nor with global health-related QoL. Anyway, the frequency of hot flushes was associated with poorer emotional function, poorer body image, anxiety and sleep problems. Patients, in general, expressed a preference for non-hormonal treatments of menopausal symptoms.

Dysfunctional sleep-related thoughts and sleep inhibitory behaviors to the insomnia process were studied by Rumble et al. (2010). Analyzing sleep diaries, nighttime pain, hot flushes, dysfunctional sleep related thoughts and behaviors, fatigue and mood, the authors found that poorer sleep was related to nighttime pain and hot flushes. Dysfunctional sleep-related thoughts and sleep inhibitory behaviors showed to be antecedents of insomnia and higher levels of pain, fatigue and hot flushes; while lower levels of positive mood and dysfunctional thoughts seemed to be consequence of insomnia in this population.

Mann et al. (2011) evaluated menopausal symptoms following breast cancer treatment and the effect of CBT-I in a randomized controlled trial. The participants had attended a clinical interview, underwent a 24-h sternal skin conductance monitoring and completed some questionnaire measuring hot flushes and night sweats, mood, QoL, hot flush beliefs and behaviors, optimism and somatic amplification. Women were randomized to receive either usual care (access to clinic support) or to usual care plus CBT-I. The data from sternal skin conductance and questionnaires were collected also at 6–8 weeks post-treatment and, later at 6 months, participants filled out questionnaires regarding the use of health services and treatments, changes in lifestyle and health over the previous 4 months of treatment. Mann et al. (2011) also found that CBT-I was significantly associated with a reduction in menopausal symptoms (self-reported and physiologically measured) as well as with a significant reduction in self-reported frequency, and significant improvements in sleep quality and QoL.

In a subsequent investigation, Mann et al. (2012), using the same study design, assessed menopausal symptoms at baseline, 9 and 26 weeks after randomization. The sample of women who received CBT-I showed less frequently hot flushes and night sweats than did those receiving usual care. These therapeutic gains were maintained up to 26 weeks.

### Studies focused on the effects of CBT-I on the immunologic system

A study by Savard et al. (2005a) aimed to assess immunological effects of CBT-I for insomnia secondary to breast cancer. The women were randomly assigned to CBT-I or waiting list. Compared to the control group, women treated with CBT-I showed higher secretion of interferon-γ and a lower increase of lymphocytes at post treatment. Levels of interferon-γ and interleukin-1β increased significantly after treatment. Significant changes in white blood cells, lymphocytes and interferon-γ were found at follow-up.

### Studies focused on the effects of CBT-I on mood, fatigue, and QoL

Dirksen et al. (Dirksen and Epstein, 2008) described the efficacy of CBT-I on fatigue, mood, and QoL in a sample of women who were at least 3 months post-completion of primary cancer treatment. Women were randomly assigned to either the CBT-I group, which received stimulus control instructions, sleep restriction therapy, sleep education and hygiene, or the control group which received sleep education and hygiene only. The study protocol consisted of a 2-week pre-treatment period, a 6-week treatment period, and 2-week post-treatment period. Participants in both treatment groups completed questionnaires on fatigue, mood, and QoL at baseline. During the 2-week pre-treatment period women completed daily sleep diaries and wore a wrist actigraph each night. The subgroup receiving CBT-I had significant improvements in fatigue, anxiety, depression, and QoL. The control group who received only sleep education and hygiene also had a statistically significant increase in QoL, with a lower depression at post-treatment.

The efficacy of CBT-I on sleep but also psychological and immunological aspects was studied by Savard et al. in 2005 (Savard et al., 2005b). Two experimental conditions were considered: CBT-I and waiting list (control condition). Follow-up evaluation was carried out 3, 6, and 12 months after treatment. At pre-treatment the patients were invited to complete several questionnaires (Insomnia Interview Schedule, Structured Clinical Interview for the DSM IV, Insomnia Severity Index, Sleep Diary, Hospital Anxiety and Depression Scale, Multidimensional Fatigue Inventory, European Organization for Research and Treatment of Cancer Quality of Life Questionnaire) and to spend three consecutive nights in the sleep lab. PSG was carried out also after treatment. Pooled analyses showed a significant sleep improvement only in the treated patients which were well maintained, and even enhanced in some cases, up to 1 month after the end of the intervention. Insomnia treatment was associated to several other positive findings, including reduced use of sleep medications, decreased anxiety and depression, and improved global QoL.

A study by Matthews et al. (2012) aimed to assess the adherence to CBT-I among BCS, following primary treatment. Participants were randomly assigned to the CBT-I or the placebo group (control condition). CBT-I was conducted by individual weekly sessions. The CBT-I components used were sleep restriction, sleep hygiene, stimulus control, and cognitive therapy. Participants filled out sleep-wake diaries and questionnaires about motivation, depression, anxiety, insomnia and fatigue. During CBT-I patients showed a progressively decreasing of adherence to the suggested rise time and time in bed; on the contrary, the adherence to prescribed bedtime remained constantly high. Lower fatigue and higher baseline motivation seemed to be the factors associated with higher adherence.

To date, the largest trial of CBT-I for insomnia in BCS has been recently conducted by Savard et al. (2014) who reported a significant improvement of all subjective sleep variables and a large reduction of dysfunctional beliefs about sleep associated with both professionally administered CBT-I (PCBT-I) and video based CBT-I (VCBT-I). Although CBT-I was shown to be associated to a significantly greater decrease than the control condition of anxiety, depression, and fatigue scores, there were no significant differences between VCBT-I and the no-treatment condition (control) on these variables. CBT-I was associated with a decrease of ISI score, early morning awakenings, depression, fatigue, and dysfunctional beliefs about sleep. The remission rate of insomnia showed to be significantly higher in CBT-I, as compared to VCBT-I.

Matthews et al. (2014) examined the effect of CBT-I on sleep, daytime symptoms, and QoL in BCS. The participants were randomly assigned to CBT-I or behavioral placebo treatment. CBT-I was associated with more improved sleep efficiency and sleep latency than behavioral placebo treatment. This improvement was sustained at follow up (3 and 6 months). Participants after CBT-I showed to have less subjective insomnia, better physical and cognitive functioning, positive sleep attitudes and increased sleep hygiene knowledge.

## Discussion

This survey reviewed the literature on CBT-I in women treated for breast cancer and evaluated the efficacy of this intervention on sleep, mood, and psychological outcomes.

All of the studies included reported a clear efficacy of CBT-I in BCS. The efficacy covers several aspects including sleep, fatigue, menopausal symptoms, mood, pain, QoL, and importantly, immunological function. The improvements associated to CBT-I were clinically and statistically significant for both subjective (sleep dairies and questionnaires) and objective measures (PSG and actigraphy). These results are in agreement with the conclusions reported by Garland et al. (2014) in a systematic review of CBT-I, in patients with different and heterogeneous types of cancer (including breast cancer) and based on only 12 controlled and uncontrolled trials.

Insomnia associated with cancer is most likely multifaceted. Although it is conceivable that other mechanisms are involved (immune response, psychological reaction, personality, changes in the circadian rhythms), the recent findings suggest a mediating role for the somatic symptoms due to chemotherapy and radiotherapy side effects, such as headache, nausea and digestive symptoms, urination, and night sweats (Savard et al., 2015). Several studies on cancer patients showed that chemotherapy and radiotherapy were associated with worsening of insomnia (Thomas et al., 2010; Palesh et al., 2012; Costa et al., 2014; Savard et al., 2001). Chemotherapy seems to have an important concurrent effect on sleep dysfunction, significantly mediated by urinary symptoms, nausea, night sweats, digestive symptoms, and dyspnea. The radiotherapy has a concurrent effect on insomnia symptoms, significantly mediated by dyspnea and night sweats (Savard et al., 2015).

All patients analyzed in the reports listed in the present review completed the primary breast cancer treatment at least 1 month before being studied; it is possible to hypothesize the existence of a complex mechanism in which several elements take part, worsening, and improving insomnia.

A recent study (Ratcliff et al., 2014) examined sleep before and during chemotherapy for BCS and suggested that subjectively disturbed sleep, during chemotherapy infusion, is associated with greater fatigue, and more negative and anxious thoughts. It is probable that improving sleep in these patients might improve their mood and, consequently, QoL.

Psychological problems such as depression, anxiety and intrusive thoughts were found to be common among breast cancer patients. Jassim et al. (2015) reviewed the effects of cognitive behavioral therapy on depression, anxiety, and mood disturbances in 28 randomized controlled trials which included a total of 3940 non-metastatic BCS. CBT was associated with decreased levels of depression, anxiety, with concomitant improvement in QoL, when compared to the control groups.

It is also important to notice that fatigue is one of the most common symptoms of any neoplastic condition. Very often, cancer-related fatigue is more severe and more enduring than that of patients without cancer (Poulson, 2001). Although in the majority of cases the etiology of fatigue is unknown and the complex relationship between fatigue and sleep remains not clearly established, behavioral, and psychosocial interventions were shown to be efficacious to improving cancer-related fatigue. This is important because, notwithstanding that fatigue is a symptom common to many pathologies, in the particular clinical picture of breast cancer comorbid with insomnia, it can contribute to a mutual auto-reinforced relationship between these two conditions.

Menopausal symptoms are very frequent in breast cancer and can be due to biological aging, to hormone replacement therapy, or to treatment (chemotherapy, ovarian ablation/suppression). Menopausal symptoms have significant negative effects on sleep and QoL. Effective non pharmacological treatment can represent a therapeutic option for menopausal symptoms too. CBT in BCS has also been reported to be effective in reducing the impact of menopausal symptoms, such as hot flushes and night sweats (Chilcot et al., 2014), independently from several variables such as age, body mass index, time since breast cancer diagnosis, menopausal status at time of diagnosis, or cancer treatment (radiotherapy or chemotherapy or endocrine treatment).

CBT-I was reported to have durable effects by most of the studies (Quesnel et al., 2003; Savard et al., 2005a,b; Dirksen and Epstein, 2008; Tremblay et al., 2009; Mann et al., 2011; Savard et al., 2011; Mann et al., 2012; Roscoe et al., 2015; Savard et al., 2005a,b), in agreement with Morin et al. (Morin and Benca, 2012) who reported that CBT-I in chronic insomnia is usually followed by a persistent therapeutic effect over time, whereas patients taking only drugs tend to relapse after discontinuation.

CBT-I might trigger a virtuous cycle by improving sleep quality which, in turn, might improve mood; mood improvement could possibly be associated with better treatment adherence and reduced intake of medication for sleep disorders, reduced anxiety and, finally with better sleep.

The treatment of disturbed sleep in BCS is important because it involves young women, without cognitive impairment, and with a long-term life expectation, as in chronic insomnia not related to cancer. Moreover, people with a history of cancer have a greater risk for depression than healthy subjects (Reyes-Gibby et al., 2006).

The main limitation of the present review is the notable heterogeneity across the studies, in terms of CBT-I type (individual CBT-I, group CBT-I, VCBT-I, PCBT-I).

Despite the simplicity and availability of self-reported sleep questionnaires, daily sleep dairies, these methods can be affected by bias, misperception, overestimation or tendency to minimize the problem. Moreover, PSG remains the gold standard for the assessment of sleep disorders, allowing to evaluate objectively sleep architecture. Therefore, PSG should be preferred to subjective sleep evaluations. Nevertheless, questionnaires have been demonstrated to be a suitable clinical instrument. Following the current standards for the diagnosis of insomnia (American Academy of Sleep Medicine, 2014), PSG is not necessary but provides important information and can exclude conditions that might cause insomnia and not be perceived by the patients. In this view, PSG should be recorded in order to exclude accurately other sleep disturbances, such as restless legs syndrome or obstructive sleep apnea.

A rich research agenda may arise from the above-reviewed concepts about CBT-I in persons already treated for breast cancer and some guidelines could be suggested. More frequent follow-ups should be scheduled; the majority of the studies planned follow-ups to 12 months, but it would be interesting and important to assess sleep for longer over time.

In order to have a more complete clinical profile of the patients and to manage opportunely the treatment, a closer collaboration between specialists in sleep and oncology is desirable. Screening for sleep disturbances is advisable in routine oncology practice, given the prevalence of insomnia in cancer and its detrimental effects on the QoL of the patients.

## Author contributions

All authors listed, have made substantial, direct and intellectual contribution to the work, and approved it for publication.

### Conflict of interest statement

The authors declare that the research was conducted in the absence of any commercial or financial relationships that could be construed as a potential conflict of interest.
